# Dr. Hurd

**Published:** 1872-05

**Authors:** 


### Death of Dr. Hurd.

Dr. S. R. Hurd, well known in Evansville, Ind., as a skillful dentist, was buried April 29, 1872. He died ere we knew he was sick. We have been acquainted with the Doctor and his family for twenty years—long before he was a citizen of this place. In his demise, our city has lost a valuable and scientific citizen. His family have our sincere condolence.—*Evansville Daily Courier*.

